# Suppressive oligodeoxynucleotides synergistically enhance antiproliferative effects of anticancer drugs in A549 human lung cancer cells

**DOI:** 10.3892/ijo.2012.1755

**Published:** 2012-12-28

**Authors:** RYOHEI TAKAHASHI, TAKASHI SATO, DENNIS M. KLINMAN, TAKESHI SHIMOSATO, TAKESHI KANEKO, YOSHIAKI ISHIGATSUBO

**Affiliations:** 1 Department of Internal Medicine and Clinical Immunology, Yokohama City University Graduate School of Medicine, Yokohama 236-0004, Japan;; 2 Center for Cancer Research, Cancer and Inflammation Program, National CancerInstitute at Frederick, Frederick, MD 21702, USA;; 3 Graduate School of Agriculture, Shinshu University, Nagano 399-4598, Japan

**Keywords:** suppressive oligodeoxynucleotides, lung cancer, Akt, extracellular signal-regulated kinase, drug synergism

## Abstract

Immunosuppressive oligodeoxynucleotides (Sup ODNs) containing repetitive TTAGGG motifs reduce inflammation and, thus, may have an impact on inflammation-related tumor growth. In this study, we found a significant antiproliferative effect of Sup ODNs on the A549 non-small cell lung cancer (NSCLC) cell line compared to those treated with control ODNs (p<0.05). Sup-ODN-mediated G1 phase cell cycle arrest was achieved via inhibition of Akt and extra-cellular signal-regulated kinase 1/2 phosphorylation and the p15^INK4b^ and p27^KIP1^/retinoblastoma protein pathway. In addition, Sup ODNs induced apoptosis and enhanced apoptosis when combined with vinorelbine. In a setting similar to clinical use of multidrug chemotherapy for advanced NSCLC, these effects were investigated by using Sup ODNs in combination with conventional anticancer drugs. Sup ODNs had a significant synergistic effect with 5-fluorouracil, vinorelbine, gemcitabine, paclitaxel and irinotecan, with a mean combination index of 0.43–0.78 (<1.0 indicates synergism) in the A549 NSCLC cell line. In conclusion, our results showed that Sup ODNs have an anticancer effect and increase the sensitivity of NSCLC cells to conventional anticancer drugs by modifying Akt and the extra-cellular signal-regulated kinase 1/2 pathway. Thus, Sup ODNs may serve as a novel therapeutic strategy for NSCLC patients.

## Introduction

Lung cancer is the leading cause of cancer-related mortality in both men and women worldwide (1). It is mainly classified into small cell lung cancer (SCLC) and non-small cell lung cancer (NSCLC). As compared to NSCLC chemotherapy, SCLC shows a better response towards radiotherapy and chemotherapy. Although the platinum-based doublet chemotherapy is the first-line standard chemotherapy for advanced disease, the response rate of NSCLC is only approximately 30%, resulting in 9 months overall survival (2). The poor prognosis of lung cancer is mainly because of the characteristics of this tumor to metastasize to distant organs early in the course of the disease. These processes are mainly mediated by transmembrane receptors called integrins, which act as the bridge between the cytoskeleton and extracellular matrix proteins, and are essential elements of tumor invasion and metastasis formation (3). Our previous study provided evidence that integrin-linked kinase, integrin β1, and the activated form of Akt are mutually associated with poor prognosis in NSCLC patients (4). In addition, it is also known that extracellular signal-regulated kinase (ERK) signaling pathway is aberrantly activated in cancer, in particular by upstream activation by the epidermal growth factor receptor and the Ras small guanosine triphosphatases and then promotes proliferation, cell survival and metastasis (5,6).

Inflammatory processes are associated with the development and/or progression of cancer (7,8). Previous reports have suggested that treatment with anti-inflammatory agents may reduce host susceptibility to cancer development (7,8). Recent studies have shown that immunosuppressive oligodeoxynucleotides (Sup ODNs) containing repetitive TTAGGG motifs prevent inflammation, including arthritis, lupus nephritis, toxic shock, acute silicosis and inflammation-associated oncogenesis (9–16).

The aim of this study was to examine the effect of Sup ODNs on NSCLC cells. Sup ODNs reduced Akt and ERK1/2 phosphorylation in a dose-dependent manner, leading to cell cycle arrest and apoptosis in A549 NSCLC cell line. Moreover, this anticancer effect of Sup ODNs was amplified synergistically in combination with conventional anticancer drugs, suggesting that Sup ODNs might be clinically important in patients with NSCLC.

## Materials and methods

### ODNs and reagents

Phosphorothioate ODNs were purchased from Integrated DNA Technologies (IDT, Coralville, IA, USA). The sequence of Sup ODN was TTAGGGTTAGGGTTAG GGTTAGGG and control ODN was GCTAGATGTTAGCGT. Previous studies have shown that the effect of Sup ODNs was sequence-dependent but not length-dependent and that the length of the control ODNs did not affect activity (16). 5-Fluorouracil (5-FU) was purchased from MP Biomedicals (Irvine, CA, USA). Gemcitabine, paclitaxel, vinorelbine ditartrate (VNR), irinotecan hydrochloride trihydrate, carboplatin and cisplatin were purchased from Wako Pure Chemical Industries (Osaka, Japan).

### Cell lines and culture

Non-small cell lung cancer cell line: A549 (p16-null, wild-type p53) cells were purchased from the American Type Culture Collection (Manassas, VA, USA) and maintained in RPMI-1640 medium (Sigma-Aldrich, St. Louis, MO, USA) supplemented with 1% (v/v) GlutaMAX (Invitrogen, Carlsbad, CA, USA), 1% (v/v) penicillin-streptomycin (Invitrogen), 10% (v/v) fetal bovine serum (Equitech-Bio, Ingram, TX, USA). Normal human bronchial epithelial (NHBE) cells were purchased from EIDIA (Tokyo, Japan) and maintained in Airway Epithelial Cell Growth Medium (PromoCell, Heidelberg, Germany). Single-cell suspensions were allowed to attach to the plate over 24 h in 6- or 96-well plates (Sumitomo, Osaka, Japan). Phosphorothioated ODNs were added to culture 1 h before administration of anticancer drugs.

### Cell viability assays

Anticancer drugs and/or ODN-mediated cytotoxicity was assessed using MTT [3-(4,5-dimethylthiazol-2-yl)-2,5-diphenyltetrazolium bromide] assay as previously described (15). Cells were seeded in 96-well plates at a density of 4,000 cells/well and allowed to adhere for 24 h. The cultures were then exposed to anticancer drug with/without Sup ODNs or control ODNs (0.1, 0.3, 1, 3, 10 and 30 *μ*M) for 72 h, followed by MTT assay. Briefly, 100 *μ*l medium containing MTT (Dojindo Laboratories, Osaka, Japan; 0.5 mg/ml) was added to the adherent cells for 2 h. Non-internalized MTT was then washed away and the cells lysed by the addition of 50 *μ*l DMSO. This released the MTT internalized by viable cells. MTT concentration was measured colorimetrically and cell viability determined as the OD_570_ of treated/untreated cultures.

### Cell cycle analysis

Adherent cells were incubated with 10 *μ*M bromodeoxyuridine (BrdU) for 45 min. Adherent cells were detached with trypsin, washed in PBS and incubated with 20 *μ*l anti-BrdU-FITC for 20 min and with 2.5 *μ*l 7-amino-actinomycin D (7-AAD) for 15 min according to the manufacturer’s instructions (BrdU Flow Kit; BD Pharmingen, San Diego, CA, USA). Analysis was performed using a BD FACSCanto II flow cytometer (BD Biosciences, San Jose, CA, USA) and FlowJo v7.6.5 64-bit software (Treestar, Ashland, OR, USA).

### Apoptosis analysis

Cells were incubated with/without 3 *μ*M Sup ODNs or control ODNs in the presence or absence of 10 nM VNR from 24 to 48 h. Adherent cells were detached with trypsin, washed in PBS and incubated with 10 *μ*l FITC conjugated Annexin V and 5 *μ*l propidium iodide (PI) for 15 min in the dark according to the manufacturer’s instructions (MEBCYTO apoptosis kit; Medical and Biological Laboratories, Nagoya, Japan). Analysis was performed using a BD FACSCanto II flow cytometer (BD Biosciences) and FlowJo v7.6.5 64bit software (Treestar).

### Western blot analysis

Cells were cultured with Sup ODNs or control ODNs (0.1, 0.3, 1, 3 and 10 *μ*M) for 1, 3, 6, 12, 18 and 24 h, and then lysed in cold buffer containing 137 mM sodium chloride, 20 mM Tris, 1 mM EDTA, 50 mM sodium fluoride, 1% Triton X, protease inhibitor cocktail and phosphatase inhibitor cocktail (Sigma-Aldrich). Protein concentrations were determined using a BCA Protein Assay kit (Thermo Scientific, Rockford, IL, USA), and 10 *μ*g whole cell extract was boiled for 5 min in sample buffer. The boiled samples were run on 4–12% gradient SDS-PAGE and transferred onto PVDF membranes. Immunoblots were probed with antibody specific to Akt (pan), phospho-Akt (Ser473), phospho-Akt (Thy308), ERK, phosphor-ERK1/2, cyclin-dependent kinase 4 (Cdk4), Cdk6, cyclin D1, p15^INK4b^, p27^KIP1^, p21 (Cell Signaling Technology, Beverly, MA, USA), cyclin E (Invitrogen), Cdk2 (EMD Millipore Corporation, Billerica, MA, USA), p53 and retinoblastoma protein (pRb) (BD Pharmingen), followed by HRP-conjugated secondary antibody (Cell Signaling Technology). Signals were visualized using an enhanced chemiluminescence kit (GE Healthcare, Piscataway, NJ, USA). Blots were reprobed with anti-β-actin antibody (Santa Cruz Biotechnology, Santa Cruz, CA, USA) to normalize for protein loading.

### Statistical analysis

Statistical analyses were performed using GraphPad PRISM, version 5.01 (GraphPad Software, San Diego, CA, USA). Differences between groups were assessed using the t-test. All tests were two-sided; p-values of 0.05 were considered significant. The interaction between Sup ODNs and conventional anticancer drugs was analyzed by isobologram analysis using CalcuSyn software (Biosoft, Cambridge, UK) to determine whether the combination was additive or synergistic; a combination index (CI) <1.0 indicated a synergistic effect.

## Results

### Effect of Sup ODNs on proliferation of A549 NSCLC cell line

**Figure 1.**
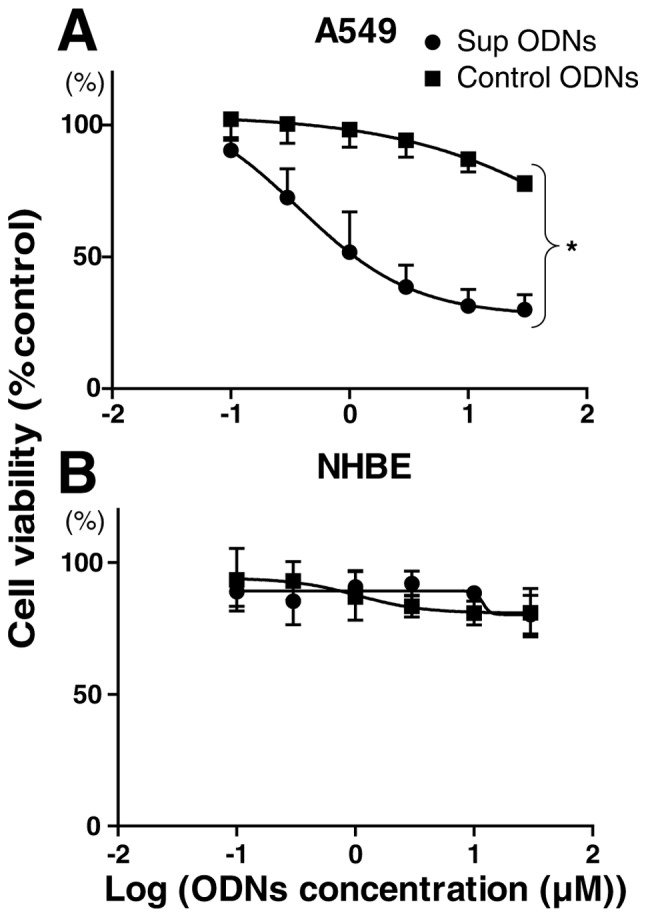
Antiproliferative effect of Sup ODNs on A549 NSCLC and NHBE cells. (A) A549 and (B) NHBE cells were treated with 0.1–30 *μ*M Sup ODNs or control ODNs for 72 h. Cell viability was evaluated using the MTT assay. Results represent mean ± SD change in cell viability from three independent experiments; ^*^p<0.05.

A549 NSCLC cell line was cultured for 3 days with 0.1–30 *μ*M Sup ODNs or control ODNs and evaluated for viability by MTT assay. There was a significant and dose-dependent reduction in viability of A549 cells treated with Sup ODNs when compared to control ODNs (Fig. 1A; p<0.05). The mean 50% inhibitory concentration (IC_50_) value for A549 cells was 2.5 *μ*M. Normal human bronchial epithelial cells treated with high concentrations of Sup ODNs (10–30 *μ*M) showed no significant reduction in viability compared to those treated with control ODNs or medium alone (Fig. 1B).

### Induction of G1 phase arrest via expression of p15^INK4b^ and p27KIP1

**Figure 2.**
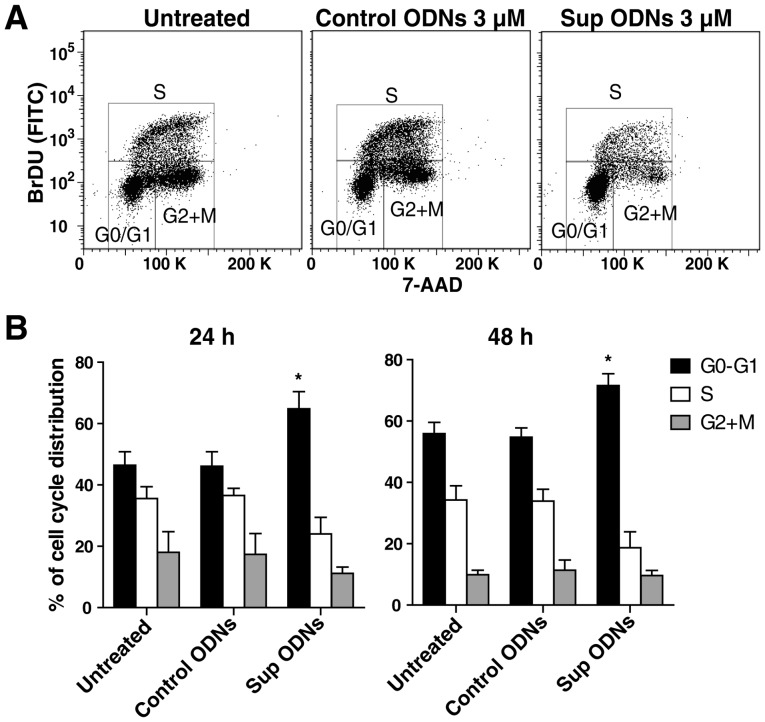
Effect of Sup ODNs on NSCLC cell cycle. A549 cells were treated with 3 *μ*M Sup ODNs or control ODNs from 24 to 48 h. Cells were stained with 7-AAD and treated with BrdU as described in Materials and methods section. (A) Representative data show the distribution of cells at each stage of the cell cycle determined by flow cytometric analysis. (B) Results show mean ± SEM from four independent experiments, performed as described above. ^*^p<0.05 when compared to cultures treated with control ODNs.

A549 cells were incubated with 3 *μ*M Sup ODNs or control ODNs. The effect of this treatment on progression through the cell cycle was analyzed by flow cytometry (Fig. 2A). Sup ODNs treatment led to ∼20% increase in the number of cells in G0/G1 phase, whereas the frequency in S phase fell by 10–15% (Fig. 2B). These findings are consistent with Sup ODNs inducing cell cycle arrest in the G1 phase.

**Figure 3.**
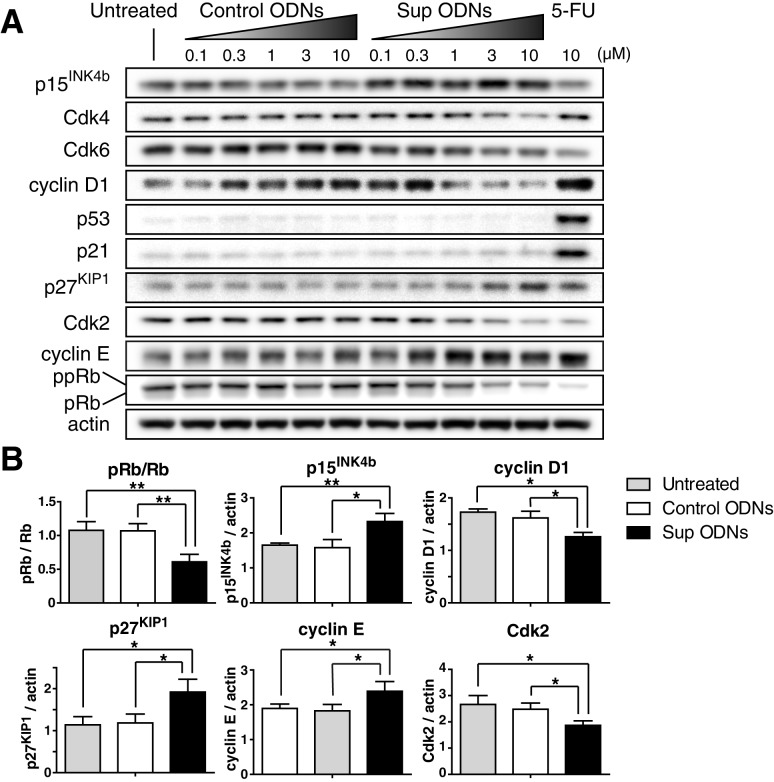
Effect of Sup ODNs on cell-cycle-related protein expression by A549 cells. (A) Expression of cell cycle-related protein was analyzed in A549 cells treated with 0.1, 0.3, 1, 3, 10 *μ*M Sup ODNs or control ODNs for 24 h, by western blot analysis. A549 cells treated with 5-FU 10 *μ*M were used as a positive control of p53 and p21 (42). (B) Band intensities of A549 cells treated with/without 3 *μ*M Sup ODNs or control ODNs for 24 h were quantified by densitometric analysis. The band intensities were normalized to actin of the corresponding lane and the signal for pRB of each lane was normalized to total RB of the same lane. Results represent the mean ± SD of three independent experiments. ^*^p<0.05, ^**^p<0.01 when compared to cultures untreated or treated with control ODNs.

Expression of cell cycle-related proteins was then analyzed by western blot analysis. Results showed that Sup ODNs increased expression of Cdk inhibitors including p15^INK4b^ and p27^KIP1^, therefore increased expression of cyclin E and reduced expression of Cdk2, cyclin D1 and retinoblastoma protein (pRb) phosphorylation in a dose-dependent manner (Fig. 3A). As shown in Fig. 3B, these changes were found at significant level in cells treated with 3 *μ*M Sup ODNs for 24 h by densitometric analysis of band intensities. These results were similar to previous reports of induced G0/G1 arrest via Cdk inhibitors including p21 and p27^KIP1^(17,18). In contrast, Sup ODNs did not increase expression of p53 and p21. Reduction of pRb phosphorylation is a key factor of G1 phase cell cycle arrest (19,20), therefore, these results demonstrated that Sup-ODN-mediated G1 phase of cell cycle arrest was achieved via the p15^INK4b^ and p27^KIP1^/pRb pathway.

### Effect of Sup ODNs on Akt and ERK1/2 phosphorylation

To clarify the mechanism of action of Sup ODNs, the screening was performed by using a Human Phospho-Kinase Antibody Array (R&D Systems, Minneapolis, MN, USA). A549 cells was cultured for 16 h under serum-starved conditions and then treated with 3 *μ*M Sup ODNs, or untreated for 1 h in normal medium. The expression of phosphorylated Akt and ERK1/2 was increased on untreated cells. In contrast, Sup ODNs reduced the expression of them (data not shown).

### Confirmation of the effect of Sup ODNs on Akt and ERK1/2 phosphorylation

**Figure 4.**
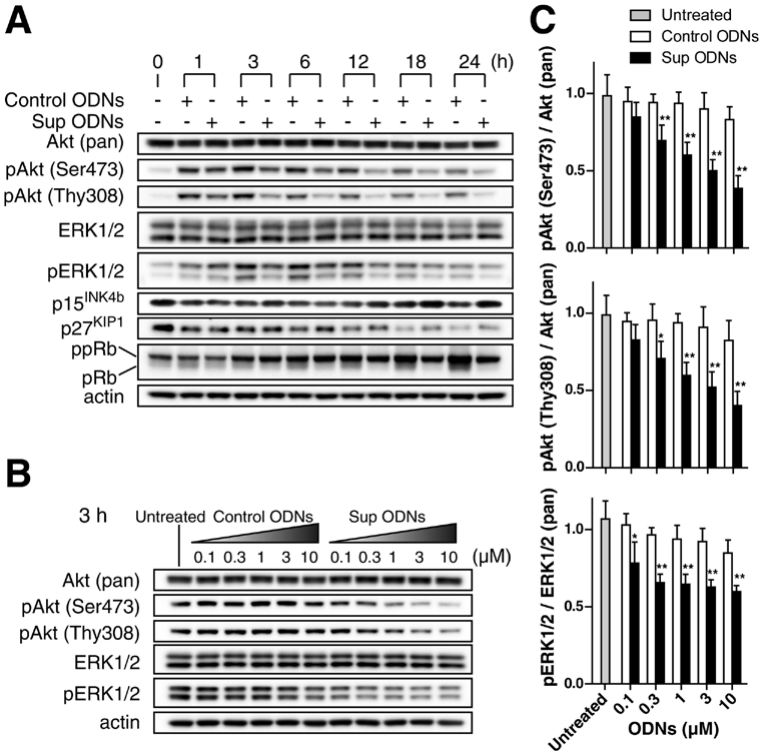
Effect of Sup ODNs on Akt and Erk1/2 phosphorylation by A549 cells. (A and B) Expression of phosphorylated Akt, phosphorylated ERK1/2, p15^INK4b^, p27^KIP1^ and pRB was analyzed in A549 cells treated with 3 *μ*M Sup ODNs or control ODNs for the indicated times, by western blot analysis. Actin was chosen as the loading control in all blots. Results are representative of at least three independent experiments. (C) Densitometric analysis of band intensity representing the mean ± SD of three independent experiments. ^*^p<0.05, ^**^p<0.01 when compared to cultures treated with control ODNs.

A549 NSCLC cell line was cultured for 16 h under serum-starved conditions to dephosphorylate Akt and ERK1/2, and then treated with increasing concentrations (0.1–30 *μ*M) of Sup ODNs or control ODNs for up to 24 h in normal medium. Western blot analysis revealed that cells treated with 3 *μ*M Sup ODNs reduced Akt phosphorylation (at both Ser473 and Thy308) and ERK1/2 phosphorylation compared to those treated with 3 *μ*M control ODNs (Fig. 4A). The effect of Sup ODNs was confirmed by a dose-dependent reduction of Akt and ERK1/2 phosphorylation or 3 h after administration (Fig. 4B and C). No such effect was observed in cells treated with control ODNs. Treatment with Sup ODNs tended to increase the level of expression of the tumor-suppressor gene p15^INK4b^ and p27^KIP1^, particularly at 18 and 24 h (Fig. 4A). From 12 to 24 h, Sup ODNs reduced the expression of pRb, which acts as control checkpoint for the G1 phase of the cell cycle (Fig. 4A). These findings suggest that G1 cell cycle arrest might be involved in the antiproliferative activity of Sup ODNs.

### Effect of combining Sup ODNs with anticancer drugs

**Figure 5.**
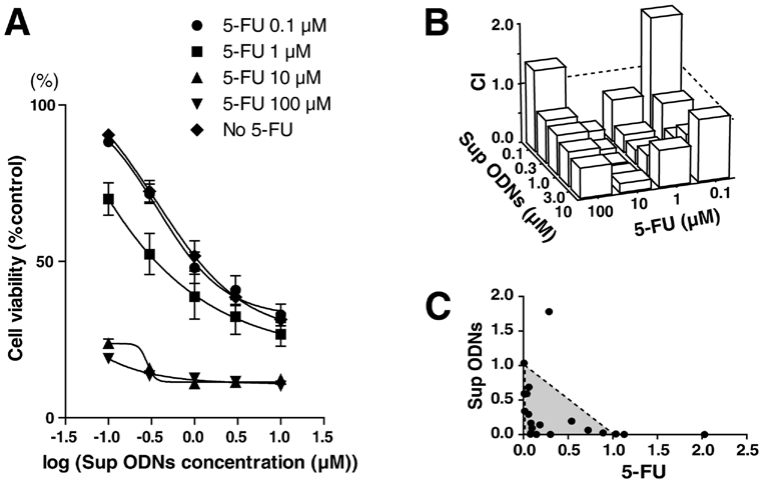
Effect of treating A549 cells with 5-FU plus Sup ODNs. (A) A549 cells were incubated with Sup ODNs for 1 h. 5-FU was added for 72 h of culture. The effect of each treatment on cell proliferation was evaluated using the MTT assay. (B) The CI for 5-FU plus Sup ODNs at various concentrations of each agent is shown. (C) Normalized isobologram for 5-FU plus Sup ODNs. Experimental data points, represented by dots located below (the shaded area), on or above the dotted line, indicate synergism, additivity and antagonism, respectively. The CI was calculated using the index-isobologram method based on the median principle developed by Chou and Talalay (21,22). CI<1 is indicative of synergy.

To examine whether Sup ODNs might interact synergistically with various drugs currently approved for the treatment of NSCLC patients, A549 NSCLC cell line was cultured with increasing concentrations of Sup ODNs plus other third-generation anti-cancer drugs (including 5-FU, gemcitabine, paclitaxel, VNR and irinotecan) or platinum-containing drugs (carboplatin and cisplatin) for 72 h and the subsequent assessment of their effects on cell viability by using the MTT assay. Combining results from multiple dose-response curves enabled us to calculate CI. The experiment used a variable-ratio drug combination design that enabled the magnitude of synergy (or antagonism) between agents to be calculated independently for each data point. Table I shows that the mean CI value for each of the combinations, except the platinum-containing drugs, ranged from 0.43 to 0.78, indicating that adding Sup ODNs to third-generation conventional anticancer drugs synergistically (defined by CI <1.0) reduced the proliferation of A549 cells. Of these, 5-FU and VNR showed favorable outcomes when combined with Sup ODNs, therefore, we further analyzed the combination effects by using these drugs. Increasing amounts of Sup ODN plus 5-FU reduced cell viability in a dose-dependent manner (Fig. 5A). Combining results from multiple dose-response curves and normalized isobolograms based on the method of Chou and Talalay are shown in Fig. 5B and C (21,22). In the normalized isobologram, experimental data points, represented by dots located below, on or above the diagonal line, indicate synergism, additivity and antagonism, respectively. As shown in Fig. 5B and C, results from combining treatment of Sup ODNs plus 5-FU indicated a favorable outcome for synergism.

### Synergistic induction of apoptosis by Sup ODNs plus anti-cancer drug

**Figure 6.**
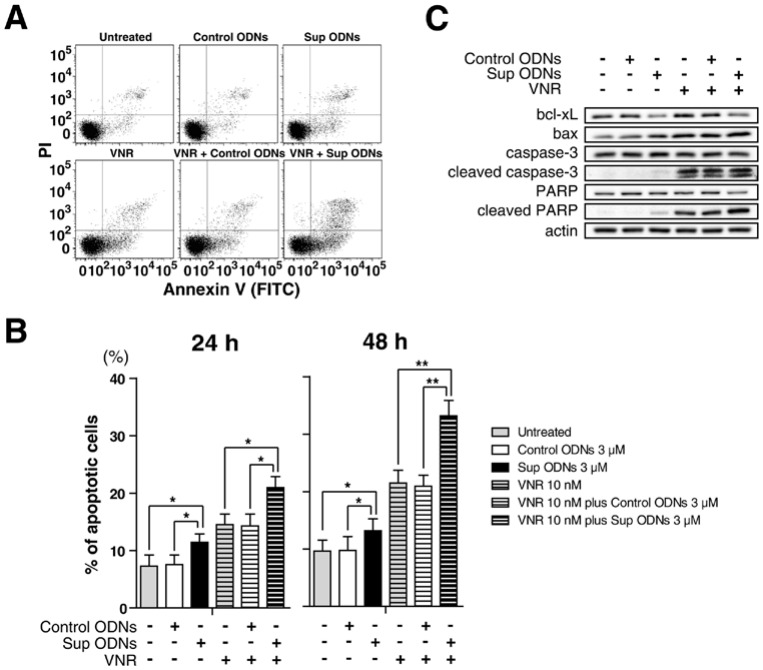
Effect of Sup ODNs on induction of apoptosis by A549 cells. (A) A549 cells were treated with/without 3 *μ*M Sup ODNs or control ODNs in the presence or absence of 10 nM VNR from 24 to 48 h. Cells were stained with FITC conjugated Annexin V and PI. Data show the distribution of apoptotic cells determined by flow cytometric analysis. (B) Results show percentage of apoptotic cells and expressed as mean ± SD from three independent experiments, performed as described above. ^*^p<0.05, ^**^p<0.01 when compared to cultures untreated or treated with control ODNs and in combination with VNR compared to cultures treated with VNR alone or VNR plus control ODNs. (C) Expression of bcl-xL, bax, cleaved caspase-3 and cleaved PARP was analyzed in A549 cells treated with/without 3 *μ*M Sup ODNs or control ODNs in the presence or absence of 10 nM VNR for 48 h by western blot analysis. Actin was chosen as the loading control in all blots. Results are representative of at least three independent experiments.

A549 NSCLC cell line was cultured with/without 3 *μ*M Sup ODNs or control ODNs in the presence or absence of 10 nM VNR from 24 to 48 h. Apoptotic cells were detected by flow cytometric analysis. Sup ODNs treatment induced apoptosis in comparison with non-treatment or control ODNs treatment. Moreover, VNR plus Sup ODNs treatment led to increased apoptotic cells synergistically (Fig. 6A and B). Western blot analysis revealed that Sup ODNs increase cleaved caspase-3, cleaved poly ADP ribose polymerase (PARP) and bax, and decrease bcl-xL that acts as pro-survival protein by inhibiting its apoptotic effect (Fig. 6C).

## Discussion

This study demonstrated that Sup ODNs inhibited A549 cell proliferation by reducing Akt and Erk1/2 phosphorylation and then increasing expression of cyclin-dependent kinase inhibitors (p15^INK4b^ and p27^KIP1^), and increased sensitivity of cells to conventional anticancer drugs.

Sup ODNs inhibit inflammatory responses and prevent the development of inflammation-dependent cancer (9–11,14,15). However, the current work is believed to be the first to document that Sup ODNs have a direct antiproliferative effect on cancer cell line. Studies involving ODNs with suppressive activity (but a different sequence than Sup ODNs) have reported that ODNs and plasmids containing telomere-derived TTAGGG sequence motifs induce apoptosis and cellular senescence via the ataxia-telangiectasia mutated (ATM) gene-p53-p21 and p16^INK4a^-pRb pathways in malignant cells (23–26). Current results examine the antiproliferative effects of Sup ODNs on A549 NSCLC cell line (p16^INK4a^-null but wild-type p53). Our results revealed that Sup ODNs did not increase the expression of p53 and p21 (Fig. 3A). Thus, our data suggest that Sup ODNs induce G1 cell cycle arrest via different pathways.

The western blot analysis results shown in Fig. 3 indicate that Sup ODNs increased accumulation of p15^INK4b^ and p^27KIP1^, consistent with G1 cell cycle arrest, loss of hypophosphorylated pRb, and increased unphosphorylated pRb (Figs. 3 and 4). The INK4 kinase inhibitors (p15^INK4b^, p16^INK4a^, p18^INK4c^ and p19^INK4d^) negatively regulate cyclin D1, D2 and D3 complexes that bind Cdk4/Cdk6 and phosphorylate pRb (19). Many human malignancies are characterized by inactivation of p16^INK4a^ or pRb, or the amplification of cyclin D1 or Cdk4 (27). The A549 cell line used in the present work was null for p16^INK4a^ but wild-type for pRb. p21 and p27^KIP1^ is a member of the Cip/Kip family of cyclin-dependent kinase inhibitors. These proteins inhibit kinase activities of pre-activated G1 cyclin E-Cdk2 and other cyclins (28). pRb exists in three general forms: unphosphorylated, hypophosphorylated and hyperphosphorylated. Freshly synthesized pRb is unphosphorylated and is present during the G0 phase of the cell cycle. Hypophosphorylated pRb is present in contact-inhibited cells during early G1. Hyperphosphorylated pRb is inactive and is present in the late G1, S, G2 and M phases of the cell cycle (29). These findings suggest that Sup ODNs induce G1 cell cycle arrest via the p15^INK4b^ and p27^KIP1^/pRb pathway rather than senescence via the ATM-p53-p21 or p16^INK4a^-pRb pathways.

We found that Sup ODNs decreased the activated form of Akt and ERK1/2 as a mechanism of increasing the expression of p15^INK4b^ and p27^KIP1^ on A549 cells. As shown in Fig. 4, the addition of Sup ODNs to cultured NSCLC cells decreased their accumulation of the activated form of the serine/threonine protein kinase Akt and ERK1/2 in a dose-dependent manner. Activated Akt phosphorylates a variety of proteins involved in critical cellular processes, including proliferation and survival (30,31). Moreover, the activated form of Akt has been linked to tumorigenesis and drug resistance in cancer cells, and correlates with poor prognosis in NSCLC (4,30,31). Similarly ERK signaling also promotes cell proliferation, cell survival and metastasis. This pathway is aberrantly activated in cancer, and the ERK pathways have attracted intense research interests (6,32). Thus, these findings suggest that Sup ODNs may be of value in the therapy of lung cancer.

Although treatment with Sup ODNs as a stand-alone agent may slow the growth of A549 NSCLC cell line, chemotherapy of patients with advanced disease typically includes multiple agents (33). Recent reports document that agents with specific molecular targets can be combined with conventional anticancer drugs to improve treatment of patients with advanced NSCLC (34,35). Thus, the potential benefit of administering Sup ODNs in combination with anticancer drugs was evaluated. As seen in Fig. 5 and Table I, Sup ODNs were found to synergize with several third-generation anticancer drugs, but not with platinum-containing drugs.

In general, agents that target microtubules (such as paclitaxel and VNR) block cell growth by inhibiting mitosis, whereas topoisomerase inhibitors (such as irinotecan) and antimetabolites (such as 5-FU and gemcitabine) are characterized by S-phase-specific cytotoxicity and induce apoptosis and G1 cell cycle arrest (36,37). Although Sup ODNs also induced G1 cell cycle arrest (Figs. 2 and 3), their activity was synergistic when combined with topoisomerase inhibitors and anti-metabolites (Table I). There are two possible explanations for this. First, topoisomerase inhibitors and antimetabolites cause DNA damage and then induce G1 cell cycle arrest via the ATM/p53/p21 pathway. This differs from the effect of Sup ODNs, which blocks the p15^INK4b^ and p27^KIP1^/pRb pathway (Figs. 3 and 4). Second, Akt inhibits cell death pathways by directly phosphorylating and inactivating proteins involved in apoptosis (38). As seen in Figs. 4 and 6, Sup ODNs decreased the activated form of Akt (phosphorylated Akt), and decreased expression of pro-survival protein (such as bcl-xL), leading to induce apoptosis. Moreover, Sup ODNs synergistically enhanced apoptosis when combined with other agents including VNR (Fig. 6).

The anticancer effect of platinum-containing drugs depends on the ability to bind covalently to DNA and subsequently to modify the structure of the DNA. Such covalent interactions result in crosslinks between adjacent nucleobases that block DNA replication and transcription, and ultimately, cell division (39). It is well known that platinum-containing drugs form preferably covalent bonds to the AG and GG sequences of DNA and ODNs (40,41). Therefore, we assume that cisplatin and carboplatin bound to Sup ODNs, which led to inhibition of effective delivery of Sup ODNs to target lesions, and thus resulting in no significant synergism for combination of platinum-containing drugs and Sup ODNs.

In conclusion, our results are believed to be the first to demonstrate that Sup ODNs have a direct anticancer effect, and increase the sensitivity of A549 NSCLC cells to conventional anticancer drugs by modifying the Akt and ERK1/2 pathway. Thus, Sup ODNs may become a novel therapeutic strategy for NSCLC patients. Studies to elucidate further the efficacy of Sup ODNs in animal models of lung cancer are planned.

## Figures and Tables

**Table I.** CI for A549 NSCLC cell line.

Anticancer drug	CI (mean ± SD at ED50)
5-Fluorouracil	0.43±0.25
Gemcitabine	0.78±0.19
Paclitaxel	0.54±0.37
Vinorelbine	0.46±0.27
Irinotecan	0.51±0.09
Carboplatin	0.94±0.11
Cisplatin	0.86±0.06

CI, combination index; ED, median effective dose.

## Abbreviations:

5-FU: 5-fluorouracil
7-AAD: 7-amino-actinomycin D
ATM: ataxia-telangiectasia mutated
BrdU: bromodeoxyuridine
Cdk: cyclin-dependent kinase
CI: combination index
ERK: extracellular signal-regulated kinase
MTT: 3-(4,5-dimethylthiazol-2-yl)-2,5-diphenyltetrazolium bromide
NHBE: normal human bronchial epithelial
NSCLC: non-small cell lung cancer
PARP: poly ADP ribose polymerase
PI: propidium iodide
pRb: retinoblastoma protein
SCLC: small cell lung cancer
Sup ODN: immunosuppressive oligodeoxynucleotide
VNR: vinorelbine ditartrate
